# Multi-Scale Simulation of GH4706 Superalloy Turbine Disk Prepared by Integral Hot Forming

**DOI:** 10.3390/ma19163373

**Published:** 2026-08-07

**Authors:** Deyu Zheng, Guoqing Zhang, Xiaoyan Sun, Jingjing Liu, Yejun Xu, Haitao Wang

**Affiliations:** 1 School of Mechanical and Electrical Engineering, Chizhou University, Chizhou 247000, China; gqzhang@czu.edu.cn (G.Z.); xiaoyansxy@czu.edu.cn (X.S.); liujingjing@czu.edu.cn (J.L.); yejunxu@163.com (Y.X.); 2 School of Materials Science and Engineering, Chongqing University, Chongqing 400044, China; 3 Brittle Material Products Intelligent Manufacturing Technology Engineering Research Center, Chizhou University, Chizhou 247000, China

**Keywords:** GH4706 alloy, microstructure, turbine disk, multi-scale simulation, level-set

## Abstract

During hot deformation of GH4706 alloy forgings, to achieve effective control over the uniformity of its microstructure, it is first necessary to establish a quantitative relationship between the microstructural characteristics of an entire hot-formed turbine disk forging and process parameters using reliable methods. Based on hot compression test results of GH4706 alloy at temperatures ranging from 950 °C to 1150 °C and strain rates from 0.001 s^−1^ to 1 s^−1^, this study developed a microstructure evolution model. Multi-scale high-precision numerical simulations were performed to predict the parameter field distribution and microstructure distribution of the turbine disk. The results reveal that the inhomogeneity of strain distribution is the primary cause of mixed grain formation. Statistical comparisons between simulation predictions at six validation points and industrial experimental data revealed the following relative deviations: 5.21% for average grain size (*AVG*), 9.65% for the standard deviation of grain size distribution (*SD*), and 5.31% for DRX fraction. Additionally, the standard deviations of prediction error for these three parameters are 3.26% for AVG, 4.06% for SD, and 3.12% for DRX fraction. These results demonstrate that the multi-scale dynamic recrystallization model developed in this study exhibits satisfactory prediction accuracy and stability. The modeling approach presented in this paper is of great significance for precisely controlling the uniformity of microstructural distribution during the hot deformation of GH4706 alloy.

## 1. Introduction

GH4706 alloy is a typical Ni-Fe-Cr alloy with excellent cold and hot formability, good creep resistance, and good oxidation resistance, which is suitable for hot-end aero-engine components, such as turbine disks, blades, and casings [1]. Studies have demonstrated that mixed grain structure is mainly caused by a low recrystallization fraction [2]. Local coarse and mixed grain structures have severe detrimental effects on the mechanical properties of turbine disks [3]. Although subsequent heat treatment can effectively regulate the microstructure to a certain extent, the microstructure after hot forming serves as the foundation and key factor determining the final performance of turbine disks. The microstructure of GH4706 alloy needs to be homogeneous and refined to ensure the service life of forgings in the extreme environments of aviation and the nuclear industry [4]. To achieve this goal, a homogenous grain structure of GH4706 alloy must be obtained through the dynamic recrystallization (DRX) process during hot deformation [5]. However, the flow behavior of GH4706 alloy is very sensitive to processing parameters, such as strain, temperature, and strain rate, which restricts suitable processing parameters to narrow hot working windows [6]. For super-large forgings of nickel-based alloys, the cost of the traditional trial-and-error method for identifying processing parameters is unacceptable. Therefore, to effectively regulate the microstructure uniformity of turbine disks, it is necessary to establish the quantitative relationship between the microstructure characteristics and process parameters of integrally hot-formed turbine disk forgings using effective methods.

Dynamic recrystallization (DRX) is the main mechanism of microstructure reconstruction in metallic materials. DRX models reveal the coupling relationship between macroscopic physical fields and microstructure evolution during deformation [7,8]. Therefore, understanding and modeling the microstructure evolution mechanism are crucial for optimizing the forming process and service performance of materials. At present, the Avrami model or modified Avrami model is widely adopted to describe the kinetic process of DRX, which are known as empirical DRX models [9]. However, these models are established based on empirical observations. Since they do not consider the nucleation mechanism and grain boundary migration driven by differences in stored energy, they cannot directly characterize and simulate DRX microstructure morphology. Accordingly, physically based microstructure models should be introduced. Common microstructure models include the phase field (PF) method, Monte Carlo (MC) method, cellular automaton (CA) method, visco-plastic self-consistent (VPSC) method, and level-set (LS) method, among others [10,11,12,13,14]. Both the level-set method and phase-field method can track microstructure evolution by directly establishing grain boundary migration equations [15]. Among them, the level-set method can directly characterize grain boundary curvature and accurately describe the nucleation and disappearance of grains, with higher computational efficiency via the level-set evolution equation [15,16,17,18,19,20]—advantages that are difficult to achieve with Monte Carlo, cellular automaton, and visco-plastic self-consistent models. Therefore, the level-set method shows great potential for simulating dynamic recrystallization.

Discontinuous dynamic recrystallization (DDRX) is a typical recrystallization mechanism for metallic materials with low/medium stacking fault energy [9]. Studies have shown that the level-set method has excellent advantages in simulating the DDRX process of face-centered cubic structures [10,11,12,13,14]. Although considerable progress has been made in DDRX simulation for large nickel-based alloy forgings, multi-scale high-precision numerical simulations targeting large forgings based on the level-set method are still in their infancy. Compared with simulations relying solely on the level-set method, this study introduces a multi-objective optimization algorithm for inverse optimization to obtain optimal parameters that minimize the error between experimental data and simulation results, thereby improving simulation accuracy. In this work, multi-scale high-precision numerical simulations are performed to predict the distribution of parameter fields and microstructures within turbine disks. Quantitative relationships between local coarse/mixed grain microstructures and local parameter fields of turbine disks are established. Furthermore, the reliability of the proposed method is verified through production tests of scaled-down forgings. This research not only provides a theoretical foundation for regulating the local microstructure homogeneity of large GH4706 alloy turbine disks by adjusting hot deformation parameters, but also proposes a reliable strategy for multi-scale high-precision simulation of heavy forgings.

## 2. Materials and Methods

**Figure 1:**
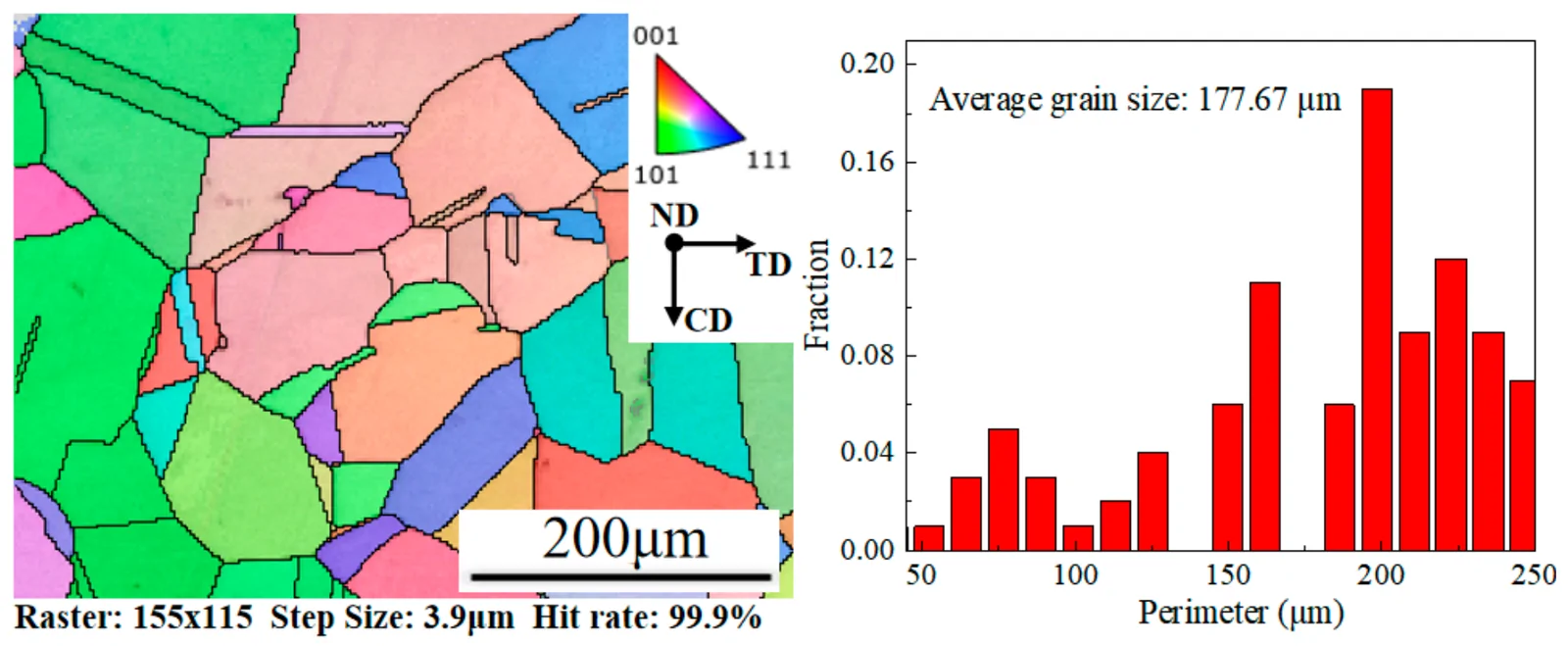
Initial microstructure of GH4706 alloy [21].

The initial test material consisted of quasi-forged GH4706 alloy after primary blooming, which represented its starting state for this study. Grain size distribution was obtained via AZtecCrystal data analysis software (AZtecCrystal 2.1), as shown in Table 1, and the weighted average grain size is approximately 177.67 μm, as shown in Figure 1. Isothermal compression tests were conducted on a computer-controlled hydraulic Gleeble-1500 thermal simulator (MTS Corporation, Eden Prairie, MN, USA). To reduce the anisotropy of the flow behavior, each specimen was heated to the target deformation temperature at a heating rate of 10 °C·s^−1^ and held for 180 s to homogenize the temperature distribution. Based on the boundary conditions of an 800 MN press, the deformation temperature was set to 950–1150 °C with an interval of 50 °C, and the strain rates were 0.001, 0.01, 0.1, and 1 s^−1^. After being compressed to a true strain of 1.2, the specimens were immediately quenched in water. True stress–true strain curves were automatically recorded by the equipment. EBSD samples were ground with silicon carbide abrasive papers up to 3000 grit, followed by electropolishing at 22 V for 20 s in an electrolyte solution consisting of 10% HClO_4_ and 90% C_2_H_5_OH. The prepared specimens were stored in anhydrous alcohol. Electron Back-Scattered Diffraction (EBSD) observations were performed on the central region of the sectioned specimens using a JSM 7800F scanning electron microscope (Siemens AG, Munich, Germany).

## 3. Construction of DRX Model Based on the Level-Set Method

### 3.1. Computational Method

**Figure 2:**
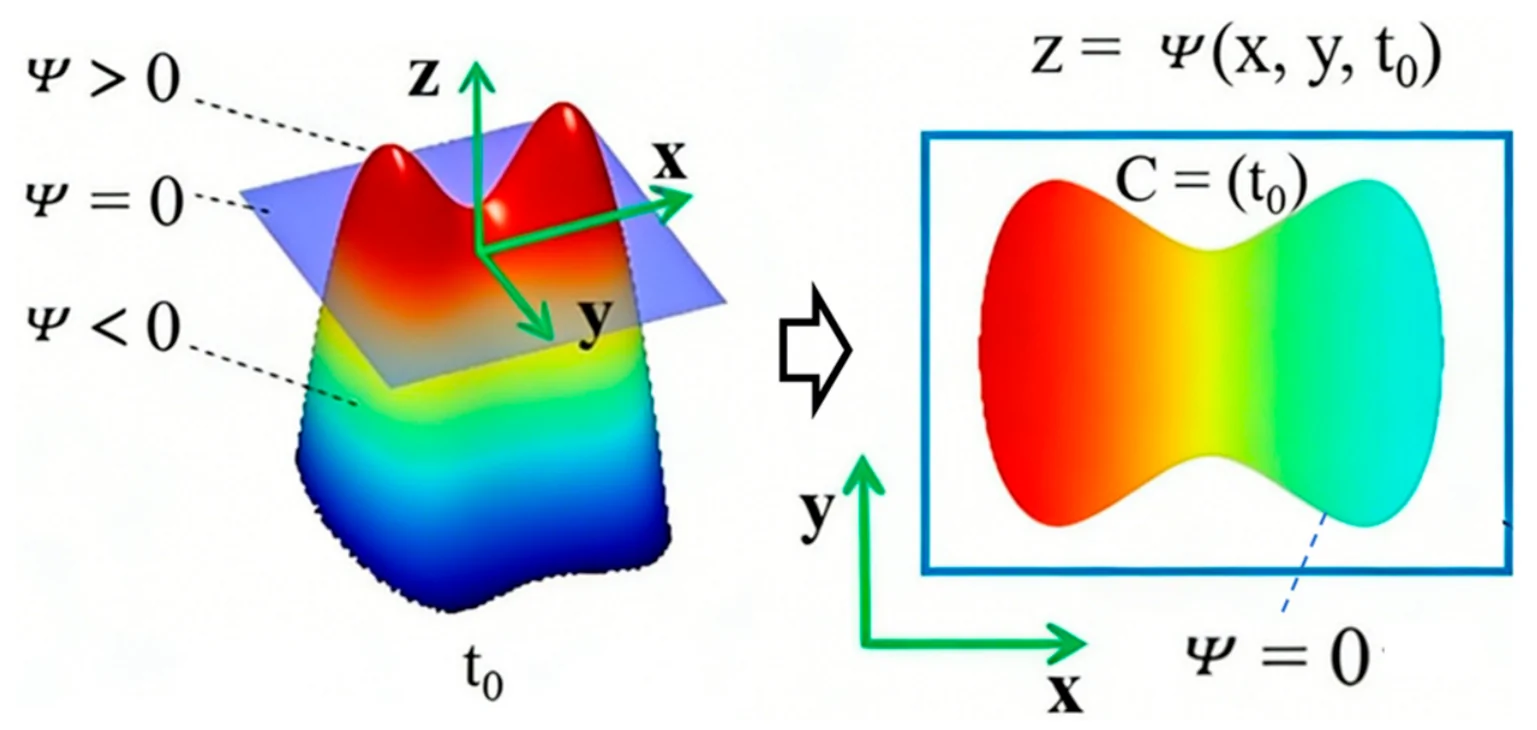
Definition of grain boundaries using level-set functions.

First, the relationship between the level-set method and the theory of curve evolution is established mathematically. A level-set function *Ψ* is defined on the domain *Ω* as the distance function to the grain boundary *Γ* of a subdomain G (i.e., a single grain). The value of *Ψ* is calculated at each node: *Ψ* > 0 inside grain G, *Ψ* < 0 outside grain G, and *Ψ* = 0 at the grain boundary of grain G. The equations describing the level-set function and grain boundaries can be expressed as [15,16,17,18,19,20]

(1) $$\left\{\begin{array}{c} \Psi_{i}\left(x,y,t\right)=\pm d\left(x,y,\Gamma \right)\;\;\;\;x\in \Omega \\ \Gamma \left(t\right)=\left\{x,y\in \Omega ,\;\;\Psi_{i}\left(x,y,t\right)=0\right\} \end{array}\right.\;\;\;\;\;\;\;\;\;\;\;\;\;\;\;\;\;\;\;\;\;$$

where *Ψ*_i_(*x, y, t*) denotes the *i*-th level-set function varying with time (*t*), and d represents the Euclidean distance. Figure 2 shows a schematic diagram of the initial grains and grain boundaries obtained by the level-set functions at time *t*_0_.

The evolution of material microstructure during deformation is described using the grain motion equation. Assume that the closed contour (*C_i_*) of the *i*-th level-set plane varies with time; the expression of the contour *C_i_*(*t*) is given by [19]

(2) $$C_{i}\left(t\right)=\left\{\left(x,y\right)|\;\Psi_{i}\left(x,y,t\right)=a\right\}$$

where *a* is the value of the level-set function at time *t*, used to judge the relationship between the level-set function and zero. Taking the total derivative of Equation (2) and applying the chain rule for composite functions yields

(3) $$\frac{d\Psi_{i}}{dt}=\frac{\partial \Psi_{i}}{\partial t}+\vec{\nabla }\Psi_{i}\cdot \frac{\partial \left(x,y\right)}{\partial t}=0\;$$

(4) $$\frac{\partial \left(x,y\right)}{\partial t}=\left(\frac{\partial x}{\partial t},\frac{\partial y}{\partial t}\right)=V\;$$

where *V* denotes the grain growth rate. Thus, Equation (3) can be rewritten as

(5) $$\frac{\partial \Psi_{i}}{\partial t}=-\vec{\nabla }\Psi_{i}\cdot V\;$$

According to the grain boundary dynamics hypothesis, grain boundary migration is driven by two types of driving forces: the “capillary effect” [17], which reduces the total grain boundary area and thus the total system boundary energy, and the *stored energy gradient* [17], under which grains with high internal stored energy are consumed by neighboring grains, thereby reducing the overall system energy. The velocity *V* can be expressed as the sum of the capillary-driven term (*V*_c_) and the stored-energy-driven term (*V*_e_) [15,16,17,18,19,20]:

(6) $$V=V_{c}+V_{e}\;$$

(7) $$V_{c}=-M\gamma \Delta \Psi_{i}\vec{\nabla }\Psi_{i}\;$$

(8) $$V_{e}=M\delta \left(\dot{\varepsilon }\right)〚E〛$$

where *M* is the grain boundary mobility; *γ* is the grain boundary energy; $\dot{\varepsilon }$ is the strain rate; and $\delta \left(\dot{\varepsilon }\right)$ is an influence factor of grain boundary migration rate caused by stored energy, a dimensionless constant defined at different strain rates and determined by multilinear interpolation in the software. 〚E〛 is the stored energy jump across the grain boundary during grain growth [16]:

(9) $$〚E〛=\tau \left(\rho_{m}-\rho_{i}\right)\;$$

where *ρ*_m_ is the dislocation density of deformed grains, and *ρ*_i_ is the dislocation density of the *i*-th equiaxed grain. *τ* denotes the dislocation line energy related to the material, which can be expressed as [17]

(10) $$\tau =\alpha \mu b^{2}\;$$

where α is the Taylor factor, generally taken as 0.5; μ is the shear modulus of the material, dependent on temperature; and *b* is the magnitude of the Burgers vector. Substituting Equations (6)–(10) into Equation (5) yields the motion equation for any one of *N* level-set functions, i.e., the motion equation of the *i*-th grain [15,19,20]:

(11) $$\left\{\begin{array}{c} \frac{\partial \Psi_{i}}{\partial t}-M\gamma \Delta \Psi_{i}+M\delta \left(\dot{\varepsilon }\right)\alpha \mu b^{2}\left(\rho_{m}-\rho_{i}\right)\vec{\nabla }\Psi_{i}=0,\;\forall i\in \left\{1,2,\ldots ,N\right\}\\ \Psi_{i}\left(x,y,t\right){|}_{t=0}=\Psi_{i}^{0}\left(x,y\right) \end{array}\right.$$

*M* and *γ* can be respectively expressed as [17]

(12) $$M=M_{0}\exp\left(\frac{Q_{m}}{RT}\right)\;$$

(13) $$\gamma =\left\{\begin{array}{c} \frac{\mu b\theta_{m}}{4\pi \left(1-v\right)}\;\theta_{i}\geq 15{}^{\circ}\\ \gamma_{m}\frac{\theta_{i}}{\theta_{m}}\left(1-\ln\left(\frac{\theta_{i}}{\theta_{m}}\right)\right)\;\theta_{i}<15{}^{\circ} \end{array}\;\right.$$

where *t* is the time step; *M*_0_ is the pre-exponential factor; *Q*_m_ is the activation energy for grain boundary migration; *R* is the gas constant; *T* is the absolute temperature; *υ* is Poisson’s ratio; *θ_i_* is the misorientation between the *i*-th grain and its adjacent grains; and *γ*_m_ and *θ*_m_ are the grain boundary energy and misorientation when the boundary becomes a high-angle boundary, respectively. To simulate the dynamic recrystallization of metals using the level-set method, a recrystallization nucleation model must be coupled to simulate grain nucleation and growth. Studies have shown that deformation temperature and strain rate significantly affect the nucleation rate [22,23,24]. A linear nucleation model is adopted in this work [25]:

(14) $$\dot{n}=K_{g}\left(T,\dot{\varepsilon }\right)\emptyset \Delta t\;\left\{\begin{array}{c} \;\emptyset =0\;\emptyset <\rho_{\mathrm{cr}}\;\\ \emptyset =1\;\emptyset \geq \rho_{\mathrm{cr}} \end{array}\right.$$

where $\dot{n}$ is the nucleation rate; $K_{g}\left(T,\dot{\varepsilon }\right)$ is the nucleation volume per unit time on any grain boundary surface satisfying the nucleation criterion, dependent on temperature and effective plastic strain rate; Δ*t* is the unit time step; $K_{g}\left(T,\dot{\varepsilon }\right)\Delta t$ is the nucleation volume per unit time on any grain boundary related to temperature and strain rate; *Φ* is the nucleation criterion function; and *ρ*_cr_ is the critical dislocation density for nucleation. There exists a Johnson–Mehl–Avrami–Kolmogorov (JMAK) kinetic relationship between the nucleation rate and the recrystallized fraction [25]:

(15) $$F=1-exp\left(-\dot{n}t^{c}\right)\;$$

where *F* is the dynamic recrystallization (DRX) fraction, and *c* is the time exponent, taken as 1. The critical dislocation density *ρ*_cr_ can be calculated by [26]

(16) $$\rho_{\mathrm{cr}}=\left[\frac{-\gamma \dot{\varepsilon }\frac{S}{M\delta \left(\dot{\varepsilon }\right)\tau^{2}}}{\ln\left(1-\frac{S}{H}\rho_{\mathrm{cr}}\right)}\right]$$

where *H* and *S* are the hardening and softening parameters, respectively, following the L–J dislocation density model, describing the evolution of critical dislocation density during hot deformation [27]. *H* and *S* are given by

(17) $$\frac{d\rho }{d\varepsilon }=H-S\rho$$

(18) $$H=H_{0}{\left(\frac{\dot{\varepsilon }}{{\dot{\varepsilon }}_{0}}\right)}^{-m}\exp\left(\frac{-mQ}{RT}\right)\;$$

(19) $$S=S_{0}{\left(\frac{\dot{\varepsilon }}{{\dot{\varepsilon }}_{0}}\right)}^{-m}\exp\left(\frac{-mQ}{RT}\right)$$

where *ε* is the strain; *ρ* is the dislocation density; ${\dot{\varepsilon }}_{0}$ is the strain rate calibration constant, set to 1 in the finite element software; *H*_0_ and *S*_0_ are the initial hardening and initial softening parameters related to temperature and strain rate, respectively; *Q* is the activation energy from the modified JMAK-type equation; and *m* is the strain rate sensitivity coefficient, reflecting the hardening trend of the material with increasing strain rate. The formula for *m* is [28]

(20) $$m={\left.\frac{\mathrm{dlg}\sigma }{\mathrm{dlg}\dot{\varepsilon }}\right|}_{T}$$

where *σ* is the flow stress. Nucleation depends on the dislocation density: when the average dislocation density in a new nucleus region reaches *ρ*_cr_ given by Equation (16), a new nucleus forms with a new average dislocation density *ρ*_0_. According to the discontinuous dynamic recrystallization (DDRX) nucleation mechanism, dislocations tend to concentrate in the grain boundary regions, and new nuclei with a critical radius *r* appear at grain boundaries, as illustrated in Figure 3.

### 3.2. Parameter Identification of Dislocation Model Parameters

Based on the true stress–strain curve, the parameters of the dislocation model in the material database GH4706.mtc were calibrated. Equation (17) was integrated to calculate *H*_0_ and *S*_0_. The dislocation density *ρ* can be expressed as

(21) $$\rho =\frac{H}{S}-\frac{C}{S}\exp\left(-S\varepsilon \right)$$

where *C* is a constant. Considering the initial conditions *ε =* 0 and *ρ = ρ*_0_, they can be expressed as

(22) $$C=H-\rho_{0}S$$

When the strain is sufficiently large, the relationship between stress and dislocation density is expressed using the Taylor formula [29]:

(23) $$\sigma =\alpha \mu b\sqrt{\rho }=\alpha \mu b\sqrt{\frac{H}{S}}$$

Substituting Equations (21) and (22) into Equation (23) yields the expression for the flow stress:

(24) $$\sigma ={\left[\sigma_{\mathrm{sat}}^{2}-\left(\sigma_{\mathrm{sat}}^{2}-\sigma_{0}^{2}\right)\exp\left(-S\varepsilon \right)\right]}^{\frac{1}{2}}$$

where *σ*_sat_ is the saturation stress and *σ*_0_ is the initial stress. Differentiating Equation (24) gives

(25) $$2\sigma \theta =S\left(\sigma_{\mathrm{sat}}^{2}-\sigma_{0}^{2}\right)\;$$

According to Equations (23) and (24), the relationship between *H* and *S* can be expressed as

(26) $$H=\frac{S\sigma_{\mathrm{sat}}^{2}}{{\left(\alpha \mu b\right)}^{2}}$$

**Figure 4:**
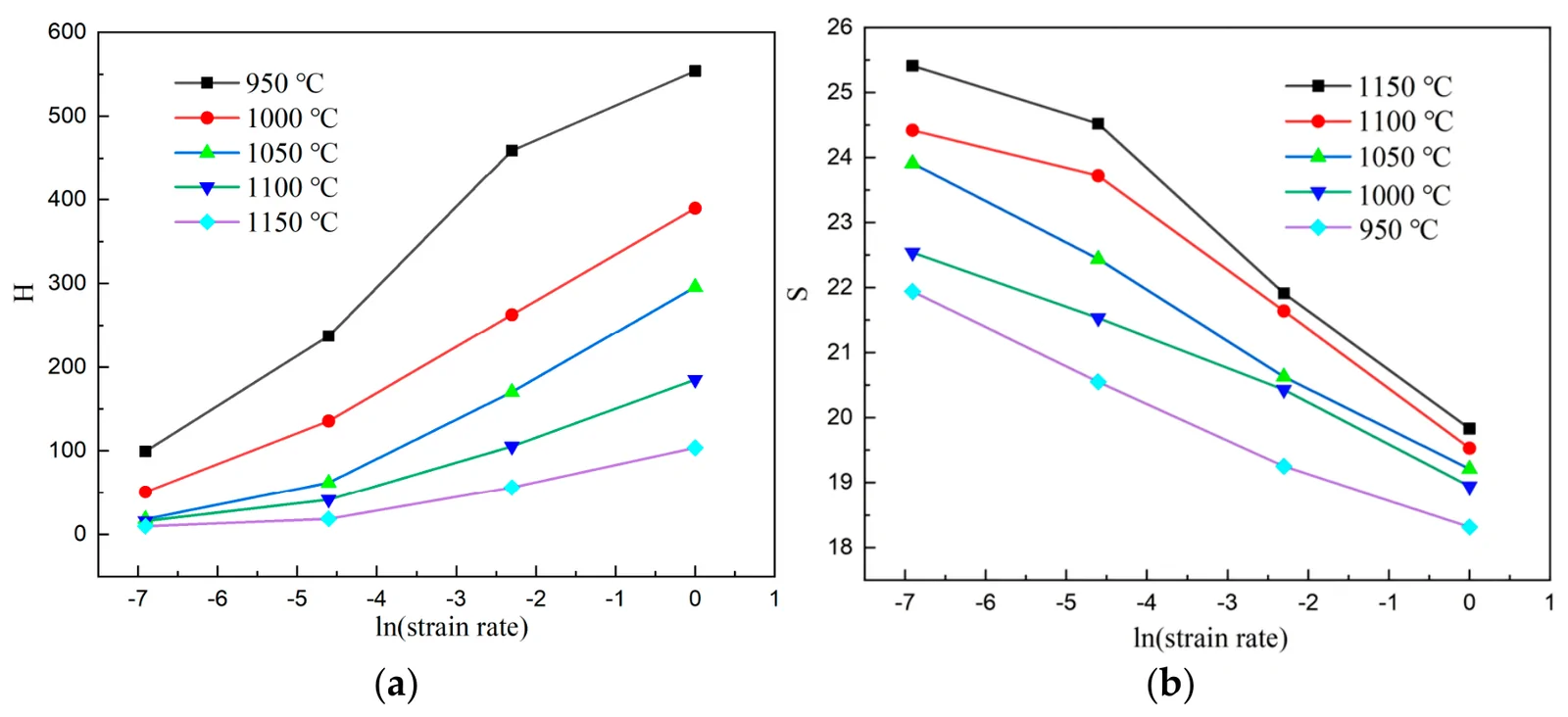
The distribution and evolution values of (**a**) *S*; (**b**) *H*.

In summary, S can be determined by plotting a *2σθ-σ*^2^ curve, where *θ* (*θ = dσ/dε*) is the hardening rate [28]. The hardening rate curve can be plotted from the true stress–strain curve. The hardening rate increases with rising stress, followed by a rapid decrease until it reaches the critical recrystallization stress. Then, the *2σθ-σ*^2^ curve was further plotted. σ_sat_ and *S* were determined from the slopes of the curves, respectively. Then, *H* was calculated according to Equation (26), and *H*_0_, *S*_0_, and *m* were obtained according to Equations (18)–(20). Figure 4 shows the distribution and evolution of *S* and *H* values. It is evident that both *S* and *H* exhibit significant regularity with variations in temperature and strain rate. Specifically, *S* exhibits a positive correlation with temperature and an inverse correlation with strain rate, whereas *H* shows an inverse correlation with temperature and a positive correlation with strain rate.

Based on this approach, the DIGIMU^®^ 4.2 software was used to develop a coupled model that characterizes the complete topological structure of grains. This model integrates the level-set distance function with the classical dislocation density model.

**Figure 5:**
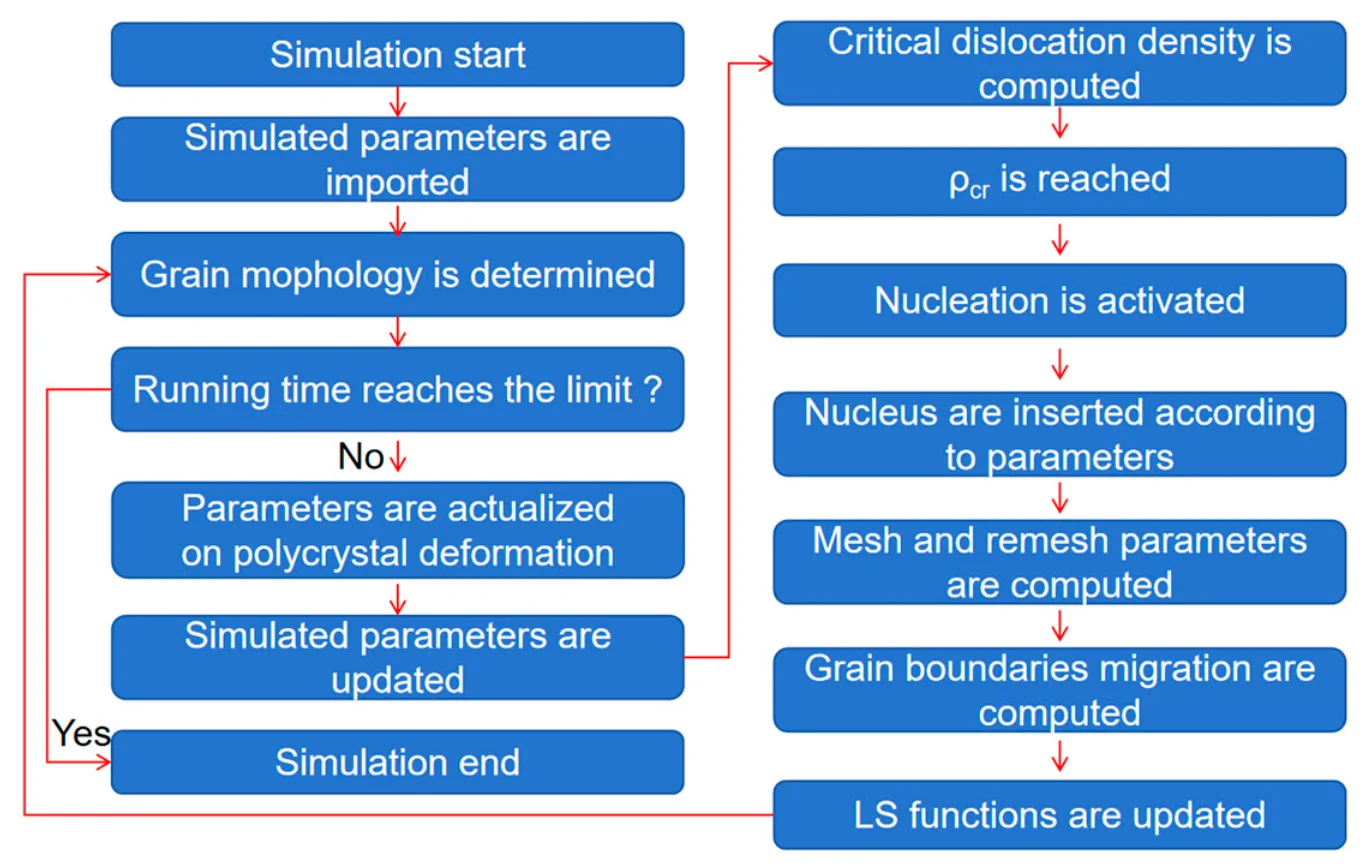
Schematic of DRX simulation using the level-set method coupled with a dislocation density model [21].

Supported by a complete computational framework for the deformation process, the following key functions were implemented: initial polycrystal generation, grain boundary representation, polycrystal deformation calculation, work hardening model, dynamic recovery model, dislocation density evolution model, nucleation model, and automatic adjustment of mesh and time step, as shown in Figure 5. At any solution step, the real-time grain morphology and instantaneous dynamic recrystallization (DRX) fraction can be obtained. Finally, through multi-step iterative calculation, the multi-field and multi-scale coupled simulation of DRX behavior and the dynamic evolution of grain morphology during hot deformation was realized.

**Figure 6:**
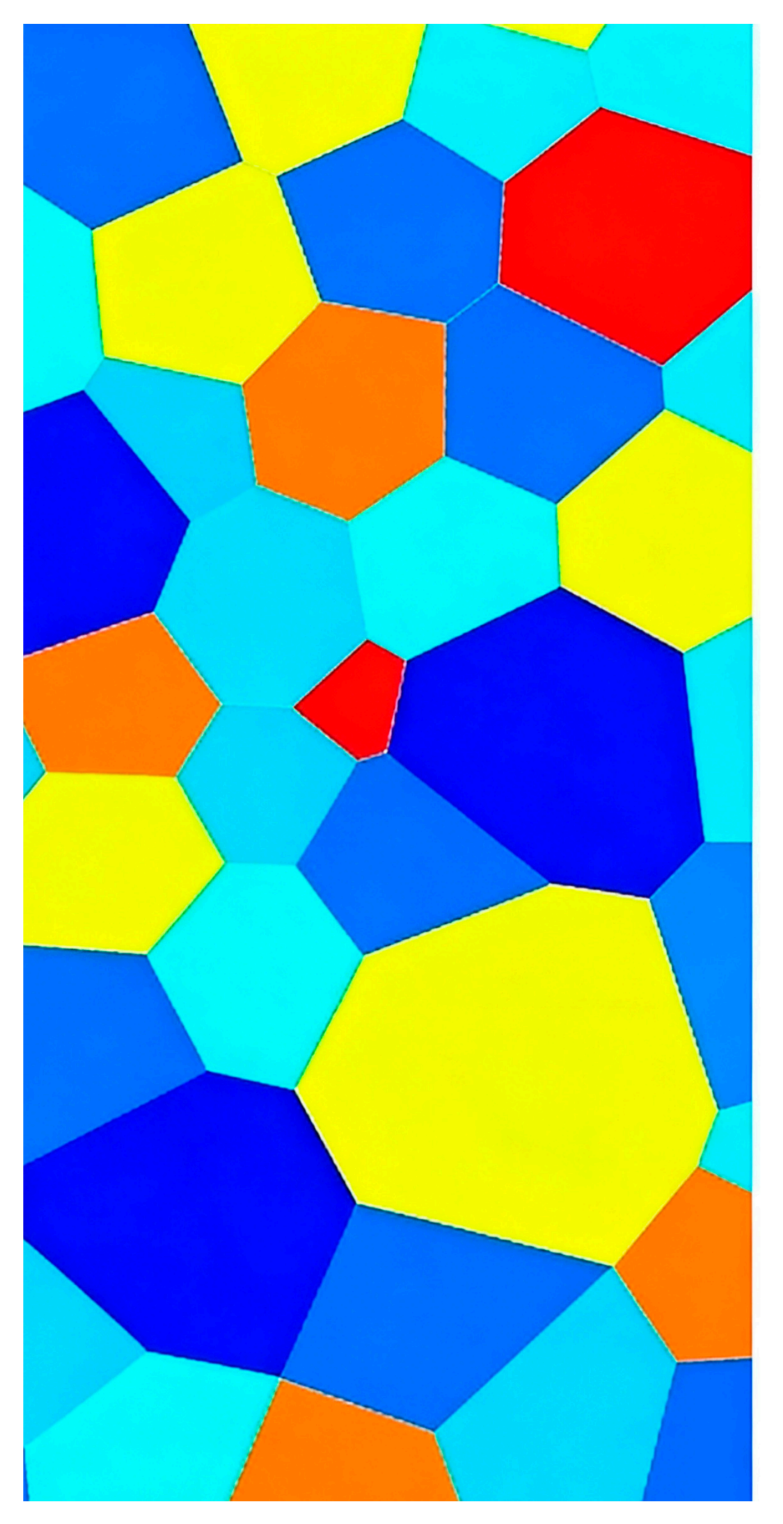
Initial microstructure model in DIGIMU [21].

The initial settings of the DIGIMU software are as follows:

(1) The simulation conditions are set as temperatures of 950–1150 **°**C, strain rates of 0.001–1 s**^−^**^1^, and a total strain of 1.2.
(2) According to the description of Figure 1, the initial average grain size is set to 177.67 μm. The initial grain size distribution data is manually entered via the Histogram option to construct the initial microstructure distribution, as shown in Figure 6.
(3) A text editor is used to input the experimental data and parameter values of the multi-field and multi-scale finite element model to build the initial material database for the GH4706 alloy, which is then selected and loaded during simulation setup.
(4) The default hardening-recovery law, threshold static recovery law, and critical dislocation density-induced nucleation initiation model are applied.

### 3.3. Identifying $K_{g}\left(T,\dot{\varepsilon }\right)$ and $\;\delta \left(\dot{\varepsilon }\right)$ Parameters Using Pareto Frontier Method

The Pareto principle mainly comprises the following three aspects [30]: (1) “Non-dominated relationship”: A solution is defined as non-dominating another solution in a multi-objective scenario if it is superior in at least one dimension and not inferior in all other dimensions. (2) “Pareto optimal solution”: A solution that cannot be improved in any single objective without compromising other objectives under given resource constraints, i.e., an irreducible solution in multi-objective optimization problems. (3) “Pareto front”: The set of all Pareto optimal solutions.

Unlike other identifiable parameters calibrated from experimental data, parameters $K_{g}\left(T,\dot{\varepsilon }\right)$ and $\delta \left(\dot{\varepsilon }\right)$ cannot be acquired via direct fitting. Specifically, parameter $K_{g}\left(T,\dot{\varepsilon }\right)$ denotes the nucleus volume generated per unit time and per unit grain boundary area once nucleation criteria are satisfied, while parameter $\delta \left(\dot{\varepsilon }\right)$ represents the additional migration velocity induced by stored energy. These two parameters require iterative reverse adjustment via continuous comparison between EBSD data and simulation results to achieve optimal values. In this study, the Pareto optimization method was adopted to implement this process. Firstly, the dynamic recrystallization (DRX) volume fraction (*F*) and average grain size (*D*) were set as optimization objectives. Subsequently, the average value of the percentage deviations between simulation and experimental results at different strains was employed as the evaluation metric, and the error functions for average grain size, *f*_1_(*K*_g_, *δ*), and DRX fraction, *f*_2_(*K*_g_, *δ*), were established, respectively:

(27) $$f_{1}\left(K_{g},\delta \right)=\frac{1}{n}\overset{n}{\underset{i=1}{\sum }}\frac{D_{p}^{i}-D_{e}^{i}}{D_{e}^{i}}$$

(28) $$f_{2}\left(K_{g},\delta \right)=\frac{1}{n}\overset{n}{\underset{i=1}{\sum }}\frac{F_{p}^{i}-F_{e}^{i}}{F_{e}^{i}}$$

where *n* is the number of experiments; $D_{p}^{i}$ is the experimental value of average grain size and $D_{e}^{i}$ is the simulated value of average grain size; $F_{p}^{i}$ is the experimental value of DRX fraction; and $F_{e}^{i}$ is the simulated value of DRX fraction.

**Figure 7:**
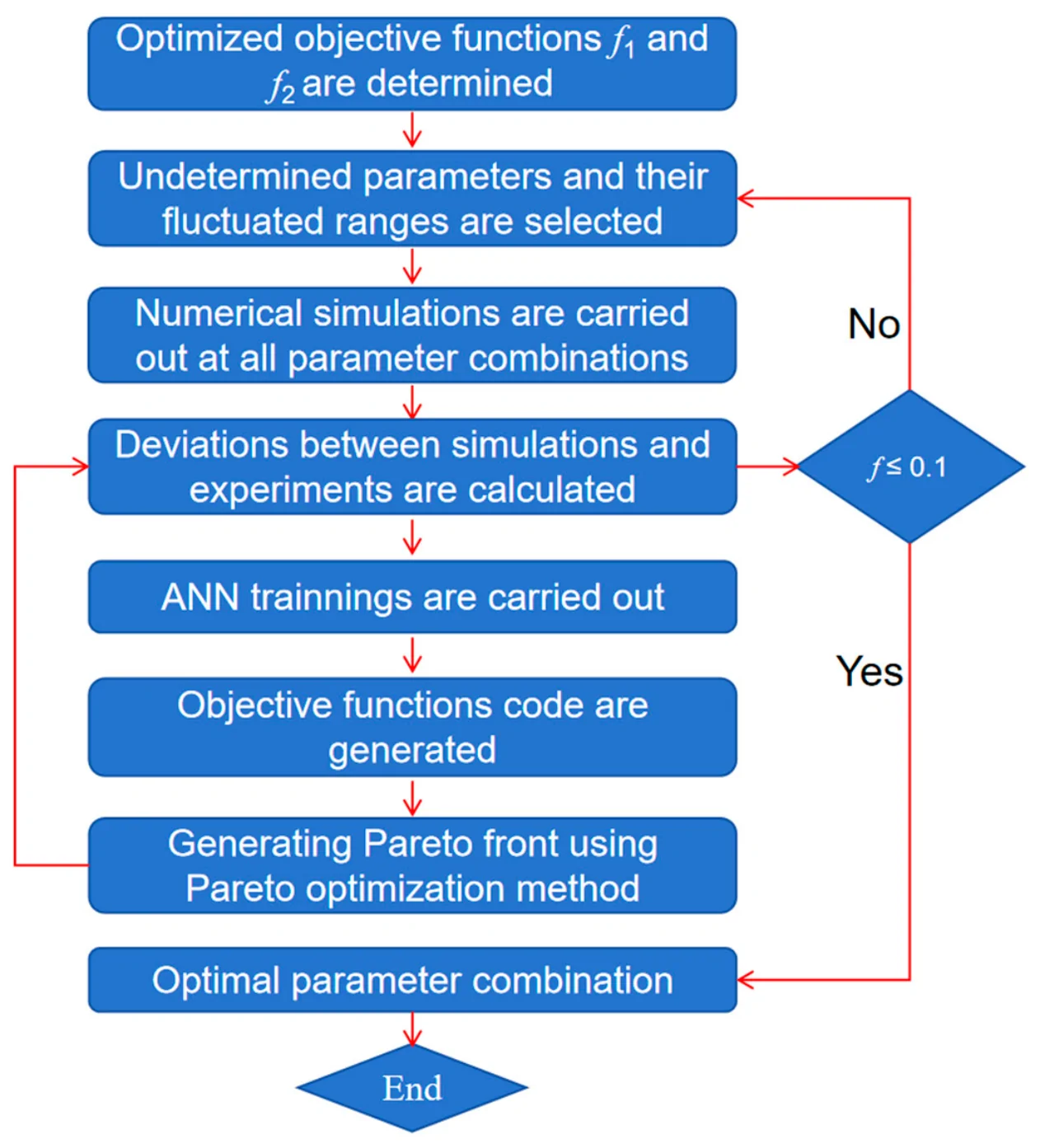
Simulation parameter identification process based on artificial neural network (ANN) and Pareto optimization method [21].

**Figure 8:**
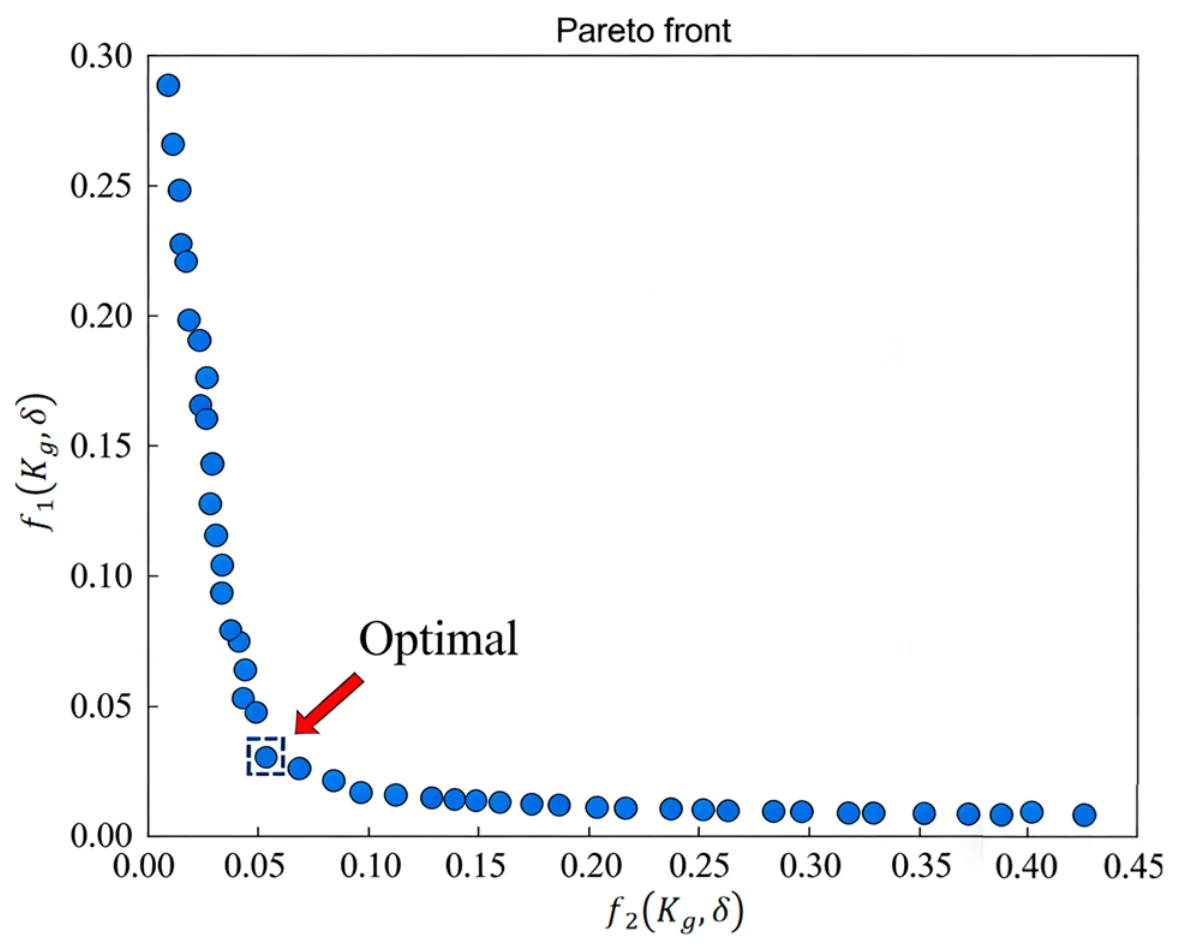
Pareto sketch map of multi-object optimization results [21].

The nucleation volume and grain boundary migration rate influencing factors were sampled within the ranges of 0~0.1 mm^3^ and 0~10, respectively, generating 40 parameter groups for simulation. The errors between the output results and experimental values were calculated using Equations (27) and (28), followed by training of a back-propagation artificial neural network (BP-ANN). Executable codes for the functions *f*_1_(*K*_g_, *δ*) and *f*_2_(*K*_g_, *δ*) were generated via MATLAB^®^ R2022b, and finally, the multi-objective Pareto front optimization in the genetic algorithm was utilized for parameter optimization. Figure 7 [21] illustrates the optimization flow based on BP-ANN training and the Pareto front method. Using the genetic algorithm toolbox in MATLAB, the Pareto fraction was set to 0.4, the population size to 100, the number of evolutionary generations to 500, and the fitness function tolerance to 1 × 10^−10^. Thereafter, the Pareto front diagram was plotted, as shown in Figure 8, with the optimized Pareto front results marked in the dashed box. The optimal parameter combination was selected from the Pareto solution set [*x*, fval] within the dashed box, and the final values of $K_{g}\left(T,\dot{\varepsilon }\right)$ and $\delta \left(\dot{\varepsilon }\right)$ were determined. The $K_{g}\left(T,\dot{\varepsilon }\right)$ and $\delta \left(\dot{\varepsilon }\right)$ parameters were updated using a text editor, establishing a calibrated material database for the GH4706 alloy. Parameter $K_{g}\left(T,\dot{\varepsilon }\right)$ is dependent on temperature and strain rate; thus, the Zener–Hollomon parameter (*Z* parameter) is introduced to establish the correlation between $K_{g}\left(T,\dot{\varepsilon }\right)$ and *Z*. In addition, the parameter $\delta \left(\dot{\varepsilon }\right)$ is governed by strain rate. In the authors’ previous research, the values of $K_{g}\left(T,\dot{\varepsilon }\right)$ and $\delta \left(\dot{\varepsilon }\right)$ corresponding to characteristic *Z* parameters were statistically analyzed. A linear relationship was found between ln*Kg* and lnZ, as well as between $\delta \left(\dot{\varepsilon }\right)$ and $\ln\dot{\varepsilon }$. Equations (29) and (30) can be obtained after fitting [31].

(29) $$\ln K_{g}=0.9919\ln Z-43.751\;$$

(30) $$\delta =0.9938\ln\dot{\varepsilon }+7.829\;$$

It should be noted that the validity of the established coupled model is only confined to the parameter range covered by the current database, i.e., temperatures ranging from 950 to 1150 °C and strain rates between 0.001 and 1 s^−1^. The microstructure of local regions in forgings with parameters beyond this range cannot be accurately simulated at present. Further expansion of the DIGIMU^®^ software database will be carried out in subsequent research to improve the generalization performance of the model.

## 4. Multi-Scale Simulation of Integral Hot Forming of Turbine Disk

In this study, to establish the relationship between the local microstructural morphology and the local parameter field of the turbine disk, and ultimately achieve homogenization of the microstructure in turbine disk forgings, the parameters *AVG* and *SD* were adopted for quantitative characterization. Specifically, *AVG* refers to the overall average grain size (including both recrystallized and non-recrystallized grains) across the entire volume, while *SD* represents the overall distribution and fluctuation of grain sizes within the entire volume.

Due to the discretization and inhomogeneous deformation characteristics of finite element simulation, the average grain size varies across each element, and this variation is independent of the number of elements. The objective functions for *AVG* and *SD* constructed based on finite element simulation are expressed as follows.

(31) $$AVG=\frac{\sum_{i=1}^{m}\int d_{i}dV_{i}}{\sum_{i=1}^{m}\int dV_{i}}$$

(32) $$SD={\left[\frac{\sum_{i=1}^{m}\int {\left(d_{i}-D\right)}^{2}dV_{i}}{\sum_{i=1}^{m}\int dV_{i}}\right]}^{\frac{1}{2}}$$

where *m* is the total number of elements in the simulated turbine disk; *d_i_* is the actual average grain size of each element in the simulation; and *D* is the overall average grain size over the whole simulated domain.

**Figure 9:**
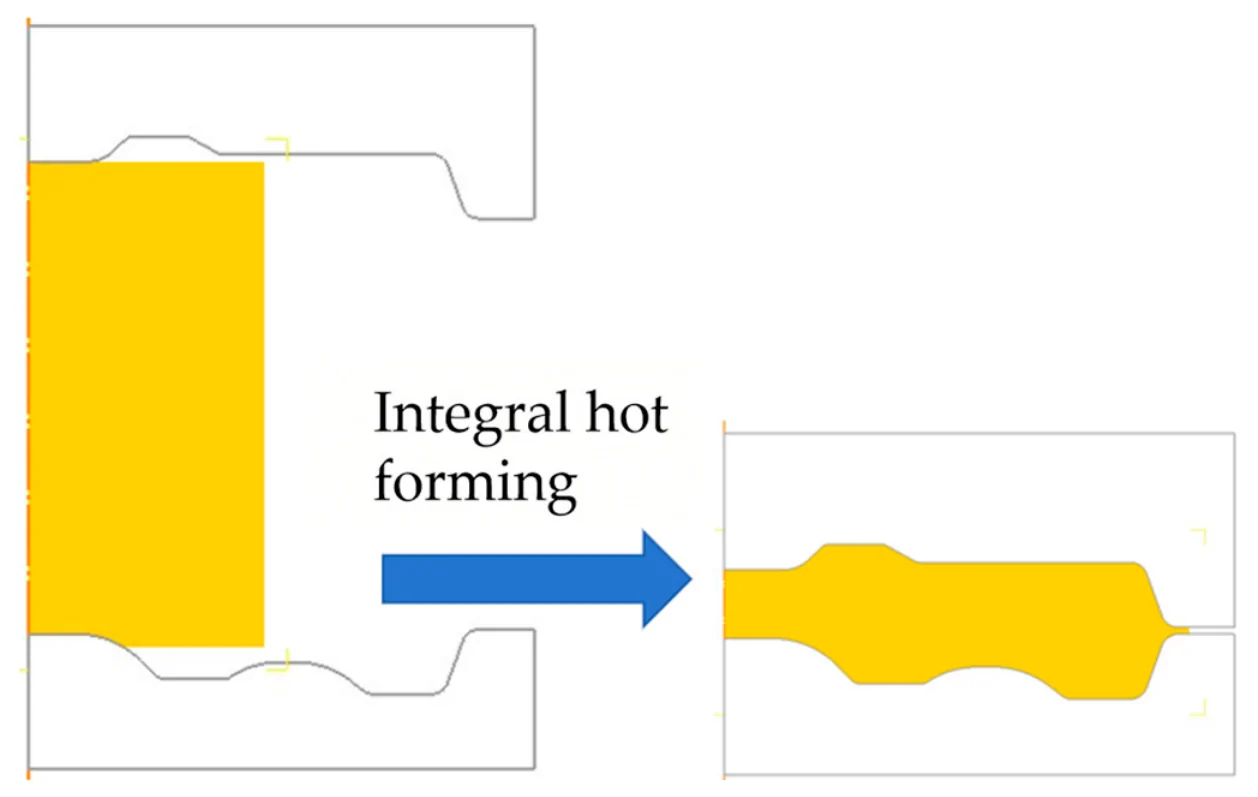
One-step integral forging process of GH4706 alloy turbine disk.

Based on the established multi-field and multi-scale dynamic coupling model of GH4706 alloy [32], which includes the GA-BP-ANN model and the dynamic recrystallization empirical model, these models were implemented into DEFORM-2D V13.1 software. A numerical simulation of the integral hot forming process was performed for a scaled GH4706 alloy turbine disk forging with a diameter of 660 mm, as shown in Figure 9.

The process conditions in the finite element simulation are as follows:

(1) Considering the high temperature and large deformation, elastic deformation was neglected. The billet was defined as plastic, while the dies were set as rigid.
(2) The friction between the billet and dies was set to shear friction with a coefficient of 0.3. The heat transfer coefficient was set to 1 N·s^−1^·mm^−1^· °C^−1^ according to the soft canned process. The ambient temperature was 20 °C, and the die temperature was 350 °C.
(3) According to previous research and equipment capacity, the deformation temperature was set at 1120 °C and the strain rate at 0.05 s^−1^ (ln*Z* = 32.794).
(4) The initial cross-section of the billet contained approximately 5000 elements, using automatic meshing technology.
(5) The optimized GA-BP-ANN model and the integrated hot forming DRX model of GH4706 alloy were embedded [32].
(6) The equilibrium equations, geometric equations, volume incompressibility condition, and von Mises isotropic yield criterion were satisfied.
(7) Microstructure evolution was taken into account during the hot forming simulation.

**Figure 10:**
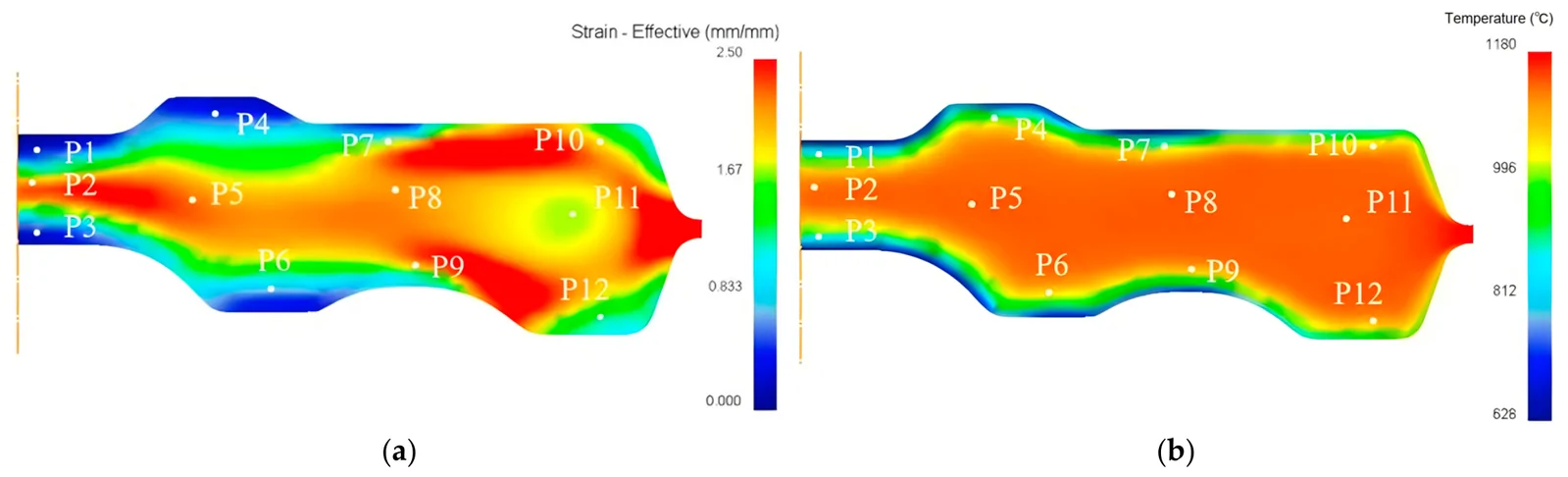

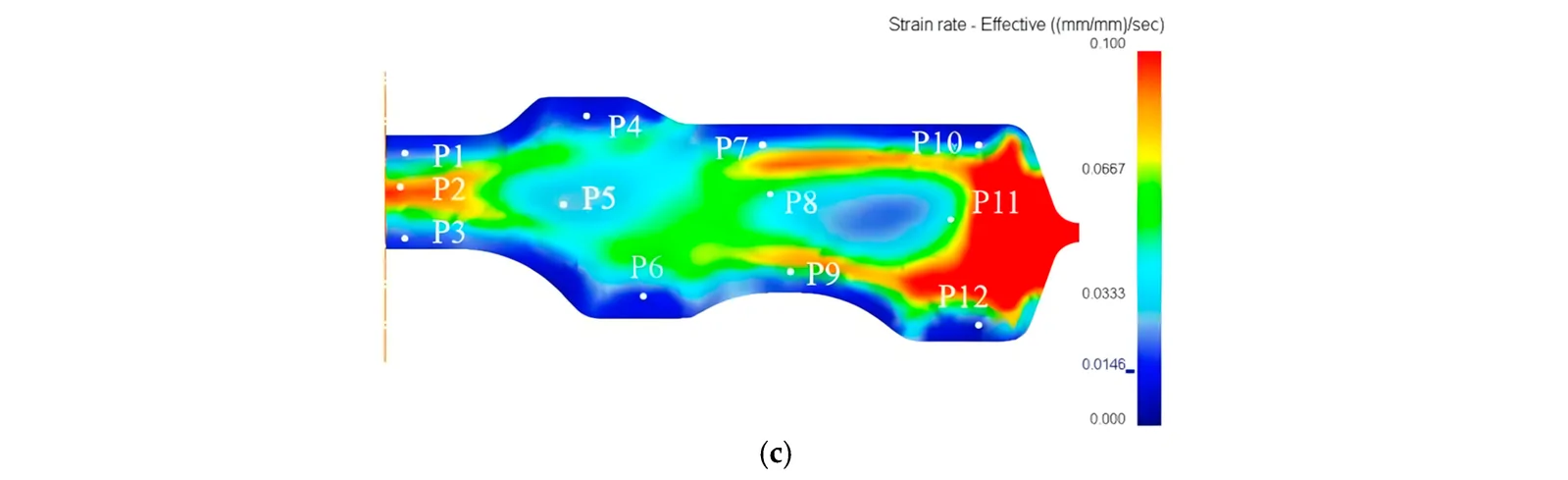
Parameter field and research points of GH4706 alloy turbine disk forging: (**a**) strain field; (**b**) temperature distribution; (**c**) strain rate distribution.

Figure 10a–c present the macroscopic field distribution nephograms of the strain field, temperature field, and strain rate field, respectively. It can be observed that the strain field and strain rate field both exhibit strong non-uniformity in their distribution. Twelve points (P1–P12) were selected across the turbine disk cross-section for investigation, forming a grid that covers the entire section. The locations of these points are defined with consideration for the subsequent surface material removal process. Macroscopic simulation can only determine the overall field distribution and the average values of local microstructural indicators, but cannot resolve the morphology of the microstructure. This means it is unable to identify the presence of mixed grains. Therefore, to characterize the mixed grain structure, further simulation of microstructural morphology is required, and the macroscopic field values obtained above serve as the necessary initial conditions for the microstructural simulation. Using the macroscopic field parameters listed in Table 2 as initial settings, the grain morphology at the 12 representative points inside the turbine disk was simulated, and this process was implemented in the DIGIMU software [19].

**Figure 11:**
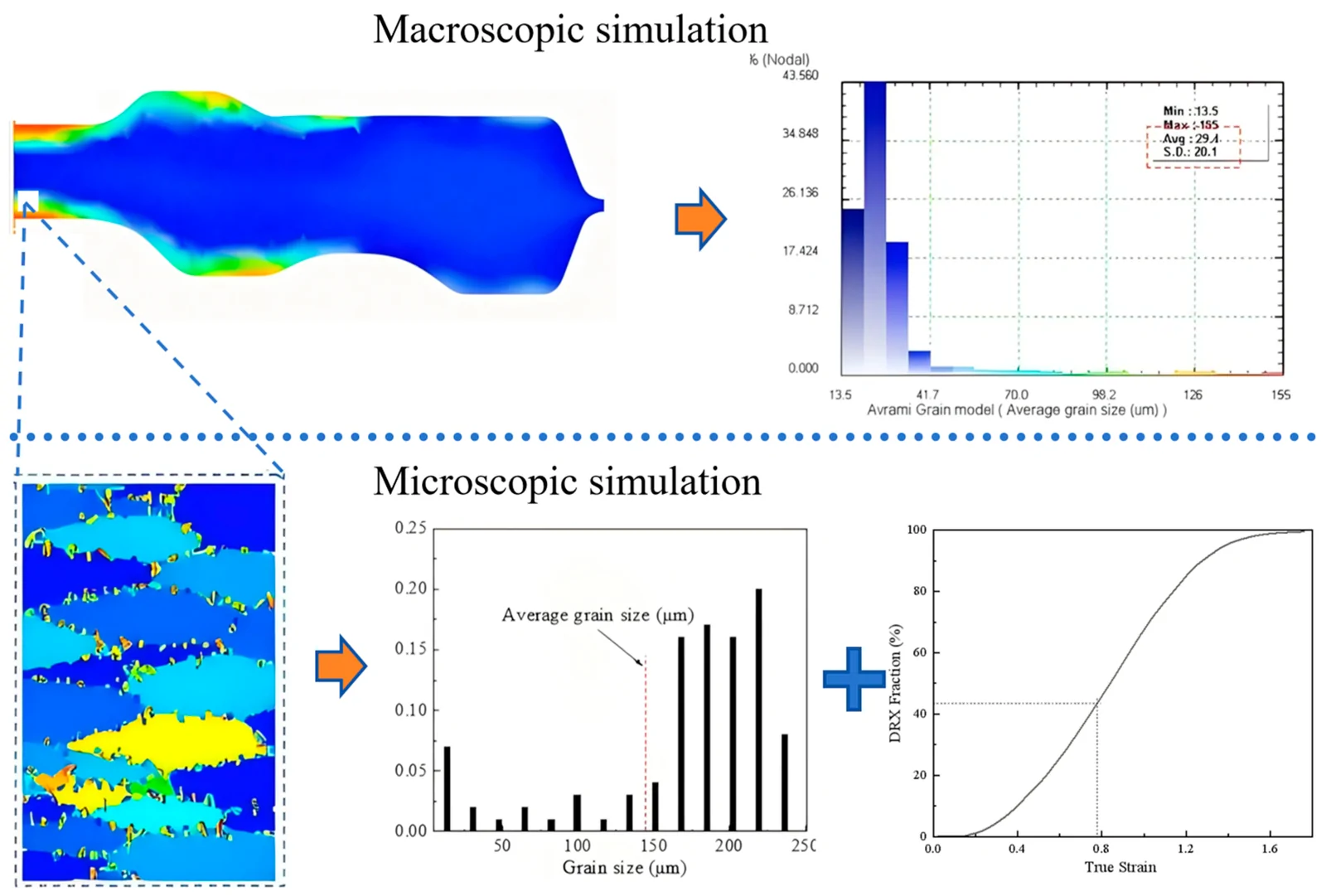
The calculation method of DRX fraction, *AVG* and *SD* of the microstructure.

The average (*AVG)* and standard deviation (*SD)* values of the twelve measurement points were obtained separately following the procedure illustrated in Figure 11. Specifically, the macroscopic *AVG* and *SD* values of the forging were extracted from the post-processing module of DEFORM software, as marked by the red dashed box in Figure 11. Microstructural simulations were then conducted to obtain grain morphology, dynamic recrystallization (DRX) fraction, and average grain size distribution. After that, the DRX fraction, *AVG* value, and *SD* value corresponding to each measurement point were calculated. Table 2 summarizes the multi-scale simulation results collected at the twelve designated points. It can be observed from the results that locations with substantial strain differences show drastically distinct *AVG* and *SD* values, and these two parameters present an obvious positive correlation. In comparison, strain rate and temperature have a weaker correlation with the *AVG* and *SD* values.

**Figure 12:**
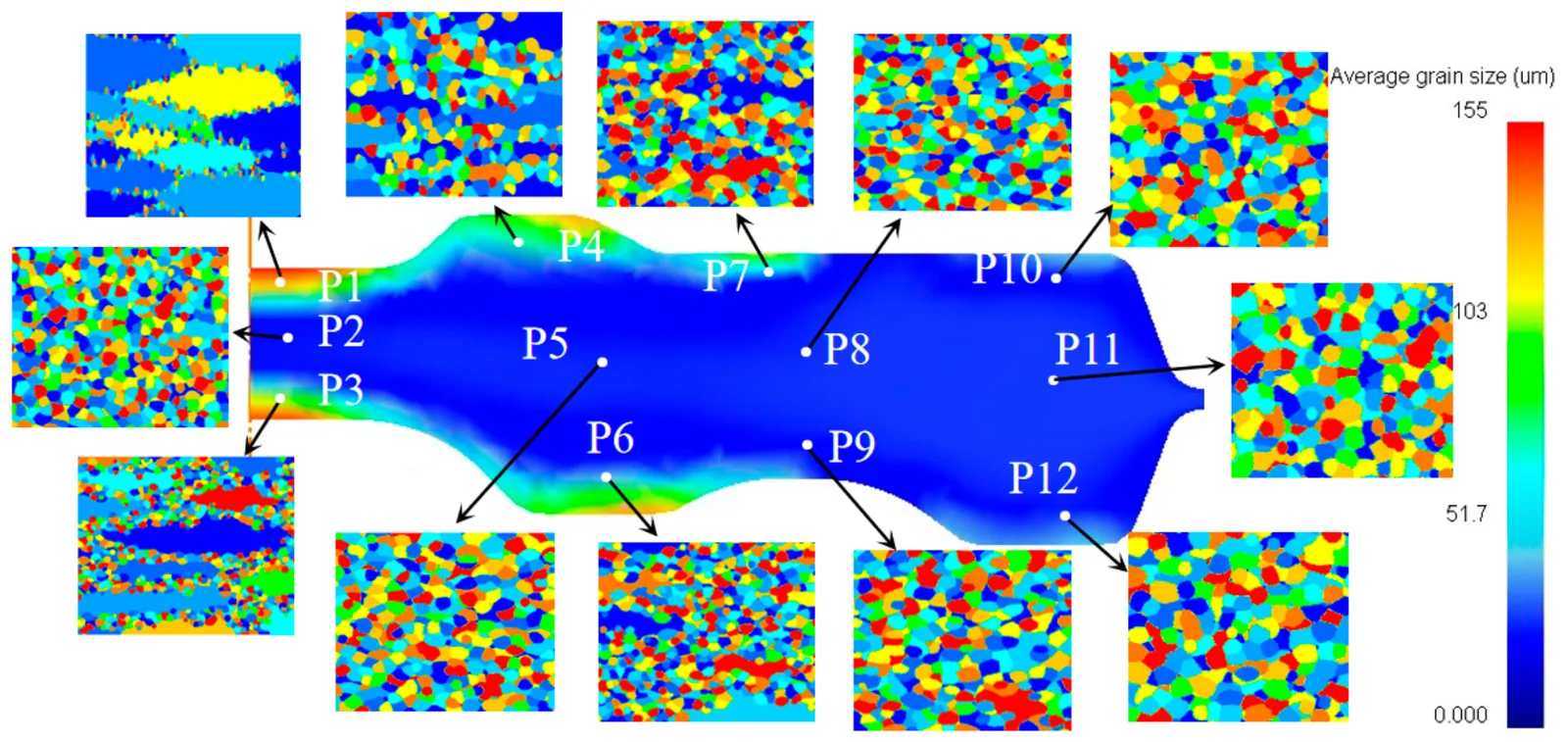
Multi-scale simulation results of the average grain size of GH4706 alloy turbine disk forging.

Figure 12 presents the simulation results predicted by the established multi-scale dynamic recrystallization model. The simulated grain morphologies at points P1–P12 were captured using fixed observation windows. Severe coarse grains and mixed-grain microstructures are observed at the hub region (points P1 and P3). Similarly, coarse and mixed-grain structures also emerge near the hub end face (points P4 and P6). In the authors’ previous research, processing map methods were adopted to verify that GH4706 alloy possesses a minimum strain threshold under specific temperature and strain rate conditions [33]. The DRX efficiency is extremely low when the strain falls below this threshold, which greatly facilitates the generation of mixed grains and coarse grains. The effective strains at points P1, P3, P4 and P6 extracted from Figure 10 (listed in Table 2) are 0.24, 0.32, 0.44 and 0.64, respectively. This demonstrates that the mixed grains and coarse grains at these four positions originate from strains lower than or close to the minimum strain threshold. Calculations based on Equations (29) and (30) yield large *AVG* and *SD* values for such mixed and coarse grain morphologies. In contrast, the effective strains at points P10 and P12 reach 1.83 and 1.19. High dislocation density is produced in these heavily deformed zones, enabling sufficient DRX processes. As a result, mixed and coarse grains are eliminated, and the microstructure evolves into fine and uniform grains, corresponding to small *AVG* and *SD* values calculated via Equations (29) and (30). From the overall perspective of the turbine disk, the hub of disk forgings always acts as a concentrated low-strain zone. This issue can be addressed by approaches such as adding a pre-forging step [2]. After one-step forming, the significant strain gradient within the turbine disk triggers severe local mixed-grain microstructures, which directly induces inhomogeneity in the microstructure and mechanical properties of the forging [3].

## 5. Production Verification

To verify the prediction accuracy of the multi-field and multi-scale coupled model, actual production verification of the forging was carried out. The process flow for the trial production of the overall hot forming of the turbine disk is as follows: hole machining → lubricant coating → canning → hot forging → rough machining → ultrasonic testing → specimen extraction → EBSD sample preparation → EBSD characterization and analysis. The die heating temperature and the upper die displacement rate were kept consistent with the simulation parameters. The diameter of the turbine disk after hot forming for production verification is 660 mm, matching the simulated shape dimensions.

The distinction between DRX grains and non-DRX grains is determined by the Grain Orientation Spread (GOS). As a quantitative parameter, GOS differentiates equiaxed grains from deformed grains by measuring intragranular misorientation. Deformation of the material induces grain distortion, which corresponds to high GOS values. In contrast, equiaxed grains experience slight or negligible deformation and thus exhibit low GOS values. This characterization method has been widely adopted by researchers to quantify the recrystallized fraction. Previous investigations [14] have demonstrated that for superalloys, grains are classified as equiaxed grains when GOS < 2°, subgrains when 2° ≤ GOS ≤ 10°, and deformed grains when GOS > 10°.

**Figure 13:**
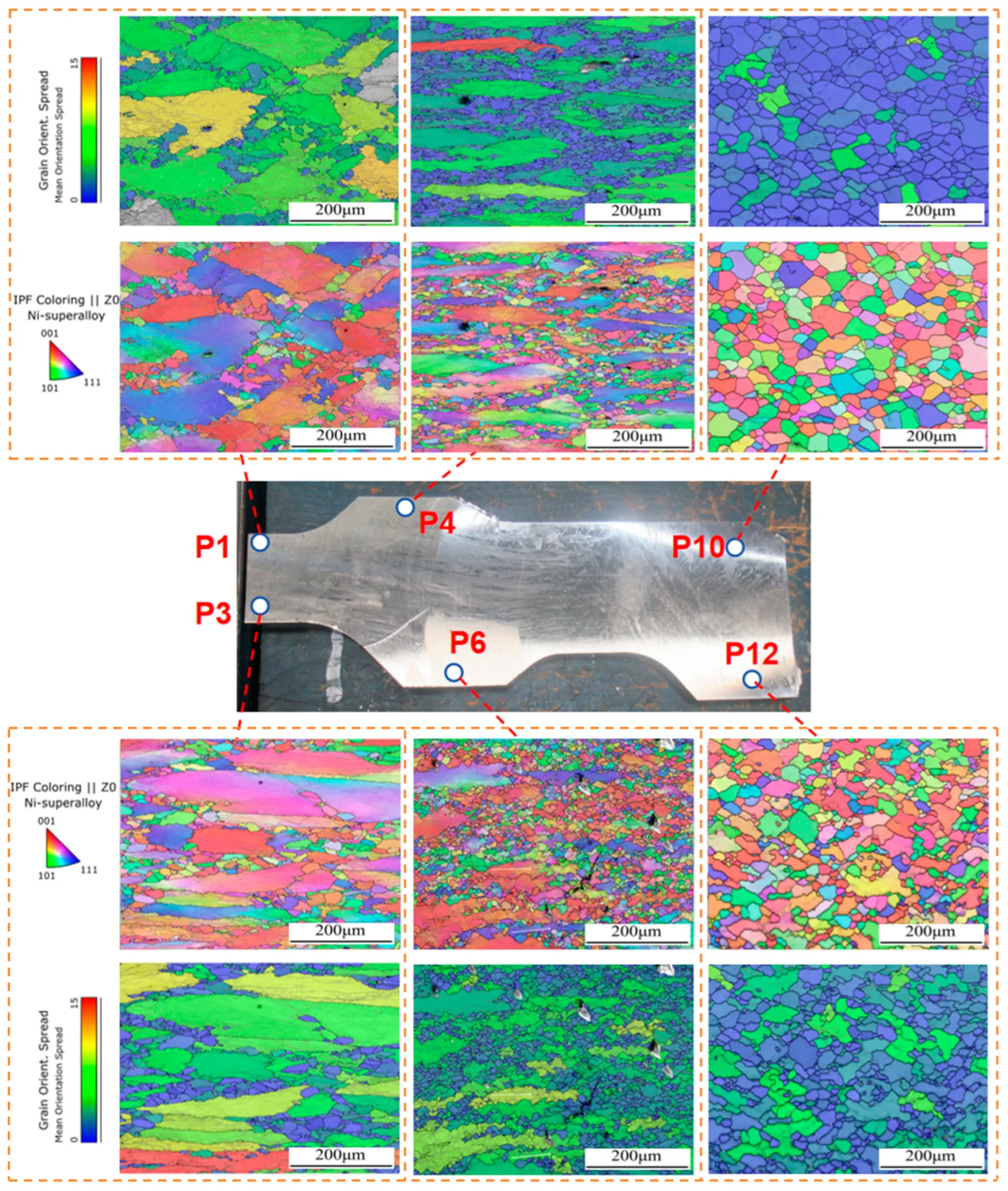
The cross-section of the workpiece and the microstructural IPF images of the specimens.

Six positions P1, P3, P4, P6, P10 and P12 were selected for experimental verification in actual production, as these six points can fully represent three typical regions of the turbine disk, namely the hub, web and rim. Figure 13 illustrates the cross-section of the workpiece and the microstructural images of specimens extracted at the six selected positions, including Inverse Pole Figure (IPF) maps and GOS maps. IPF maps allow intuitive observation of microstructural morphologies. It can be observed that the microstructures at the four points P1, P3, P4, and P6 exhibit relatively distinct mixed grain structures, whereas the microstructures at the two points P10 and P12 form well-developed dynamic recrystallized structures. A comparison with Figure 12 reveals that the simulated microstructural results for the six points show similar morphologies to the actual microstructures. Furthermore, the *AVG* and *SD* values derived from these maps can be calculated, and the observed microstructural features and statistical data are subsequently compared with simulation predictions. GOS maps are utilized to quantitatively calculate the DRX fraction of the microstructure, which is then compared with the DRX fraction output from numerical simulations. All statistical data are summarized in Table 3.

To compare the predictive capability and stability of the three constitutive models, the calculation formula for the “relative deviation (*Δ*)” is introduced:

(33) $$\Delta_{i}=\left|\frac{E_{i}-P_{i}}{E_{i}}\right|$$

(34) $$\Delta_{\mathrm{avg}}=\frac{1}{x}\overset{x}{\underset{i=1}{\sum }}\left|\delta_{i}\right|\;$$

where *Δ_i_* is the relative deviation of the *i*-th data point, *E_i_* is the experimental value of the *i*-th data point, *P_i_* is the predicted value of the *i*-th data point, *x’* is the total number of data points, and *Δ_avg_* is the average value of the relative deviation, which can effectively reflect the predictive capability and stability of the model. To provide a more intuitive quantitative evaluation of the prediction accuracy and stability of the model, the “standard deviation (*ξ*)” is introduced as a measure of the dispersion of individual points among all data, whose expression is given in Equation (33) [34]. A smaller *ξ* indicates that the relative deviation *Δ_i_* is closer to its mean value *Δ_avg_*, and vice versa. A smaller *ξ* corresponds to better predictive capability and stronger prediction stability of the model.

(35) $$\xi =\sqrt{\frac{1}{x-1}\overset{x}{\underset{i=1}{\sum }}{\left(\Delta_{i}-\Delta_{\mathrm{avg}}\right)}^{2}}$$

**Figure 14:**
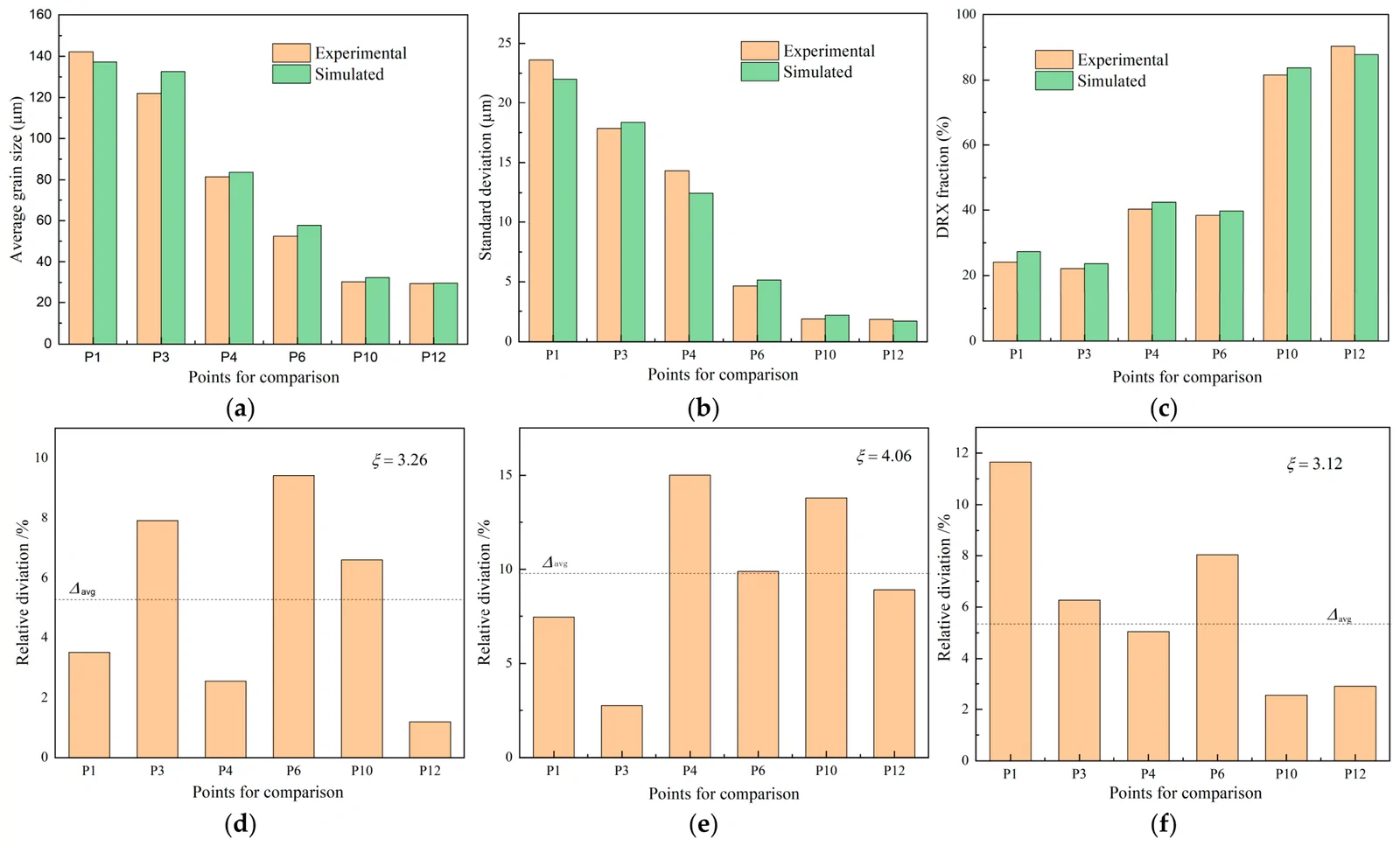
Simulated and experimental comparison of verification points: (**a**) *AVG*, (**b**) *SD*, and (**c**) DRX fraction; *ξ* values of verification points: (**d**) *AVG*, (**e**) *SD*, and (**f**) DRX fraction.

To further evaluate the prediction accuracy of the developed model, quantitative characterization and comparison between the simulated and experimental values of the *AVG*, *SD*, and DRX fraction were carried out, as illustrated in Figure 14a–c. The average deviation between simulated and experimental results was less than 10%, demonstrating that the multi-scale DRX model of GH4706 alloy established via inverse multi-objective optimization in this work possesses sufficient accuracy to meet the requirements of engineering prediction. This model can reliably reproduce the microstructure morphology, distribution, and evolution of GH4706 alloy turbine disks under various processing parameters during hot deformation. Figure 14d–f characterize the prediction stability of the model. As shown by the *ξ* values in Table 3, the average standard deviations corresponding to the three metrics (*AVG*, *SD*, and DRX fraction) are all within 10%, which verifies that the DRX model constructed in this study exhibits satisfactory prediction stability. Specifically, the sensitivity index follows the order: *SD* (*ξ* = 4.06) > *AVG* (*ξ* = 3.26) > DRX fraction (*ξ* = 3.12). This result indicates that the model is most sensitive to the prediction of the *SD* and delivers the most stable predictions for DRX fraction. The above conclusions are of great significance for the precise regulation of microstructures in GH4706 alloy forgings during hot deformation.

**Figure 15:**
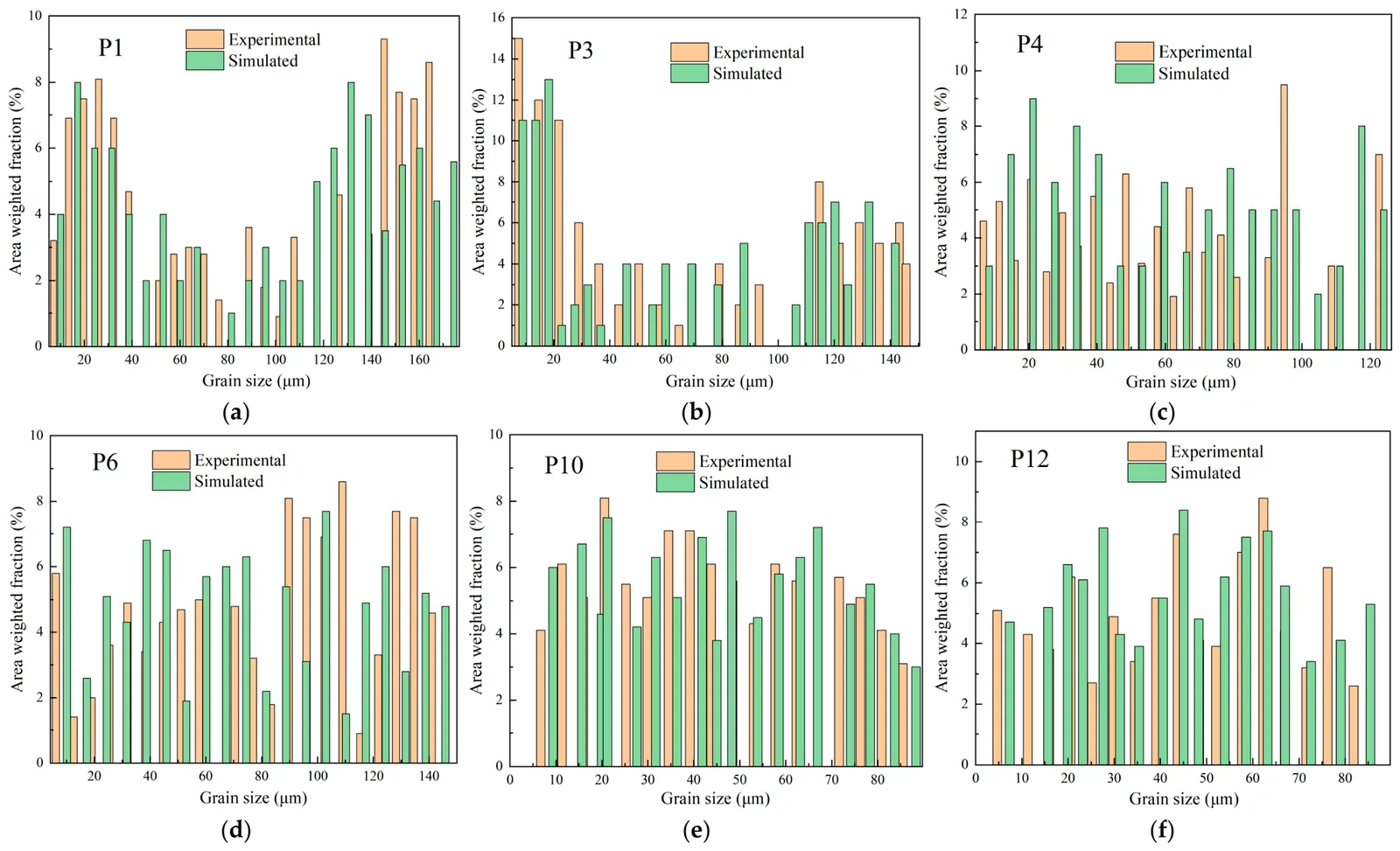
Experimental and simulated grain size distributions at different points of (**a**) P1, (**b**), P3, (**c**) P4, (**d**) P6, (**e**) P10, and (**f**) P12.

Figure 15 compares the simulated and experimentally measured grain size distributions at six observation points. As can be observed, the overall grain size distributions obtained from simulation statistics agree well with the experimental results. The grain size distributions exhibit regular patterns closely correlated with strain, as illustrated in Figure 12. As shown in Figure 15a,b, the grain size distribution displays a bimodal characteristic: high fractions at both small and large grain sizes, and a low fraction in the intermediate range. Grain sizes are mainly concentrated in the ranges of 0–20 μm and above 100 μm, indicating a low dynamic recrystallization (DRX) fraction within the microstructure. The microstructure is dominated by fine DRX grains and coarse deformed grains, which can be attributed to the low strain levels at positions P1 and P2. Figure 15c,d reveal a broader, more random grain size distribution. With increasing strain at positions P4 and P6, the DRX fraction rises; meanwhile, coarse deformed grains are refined and their volume fractions decline as DRX proceeds, resulting in a more random grain size distribution. As presented in Figure 15e,f, the range of grain sizes is markedly narrowed, and grains larger than 90 μm are no longer present. This phenomenon suggests that further strain accumulation at positions P10 and P12 drives nearly all deformed grains to complete full DRX, while the newly formed DRX grains grow moderately. Consequently, the final microstructure consists of randomly distributed grains smaller than 90 μm.

Nevertheless, limited by existing experimental verification conditions, the model established in this study is only valid for forgings with geometries similar to turbine disks, that is, large disk-shaped forgings. In future work, the methodology and framework adopted herein will be extended and validated for forgings covering a wider range of geometric configurations.

## 6. Conclusions

This paper constructs a multi-scale dynamic recrystallization model for GH4706 alloy based on the level-set method, which integrates physical mechanisms including nucleation and dislocation, using hot compression experimental data. For parameters that cannot be obtained through fitting, the Pareto multi-objective optimization method is adopted for inverse identification by minimizing the deviation between experimental and simulation results, so as to obtain the optimal solution. Comparison between simulation and experimental results shows the following:

The multi-scale simulation method based on the level-set method can clearly characterize the microstructure morphology, grain distribution, and evolution process of GH4706 alloy under different strain conditions.

The deviations between the simulated average grain size (AVG), standard deviation (SD), and dynamic recrystallization (DRX) fraction and the corresponding experimental data are 5.21%, 9.65%, and 5.31% respectively, which verifies that this multi-scale simulation method can effectively meet the engineering prediction requirements for GH4706 alloy forgings.

The standard deviations between the simulated AVG, SD, and DRX fraction and the experimental data are 3.26%, 4.06%, and 3.12% respectively, indicating that the dynamic recrystallization model established in this study has satisfactory prediction stability.

The above results demonstrate that the multi-scale dynamic recrystallization model framework constructed in this paper based on multi-objective optimization can effectively predict the microstructure evolution of GH4706 alloy forgings, which is of great significance for regulating the microstructure during hot deformation processing.

## Figures and Tables

**Figure 3:**
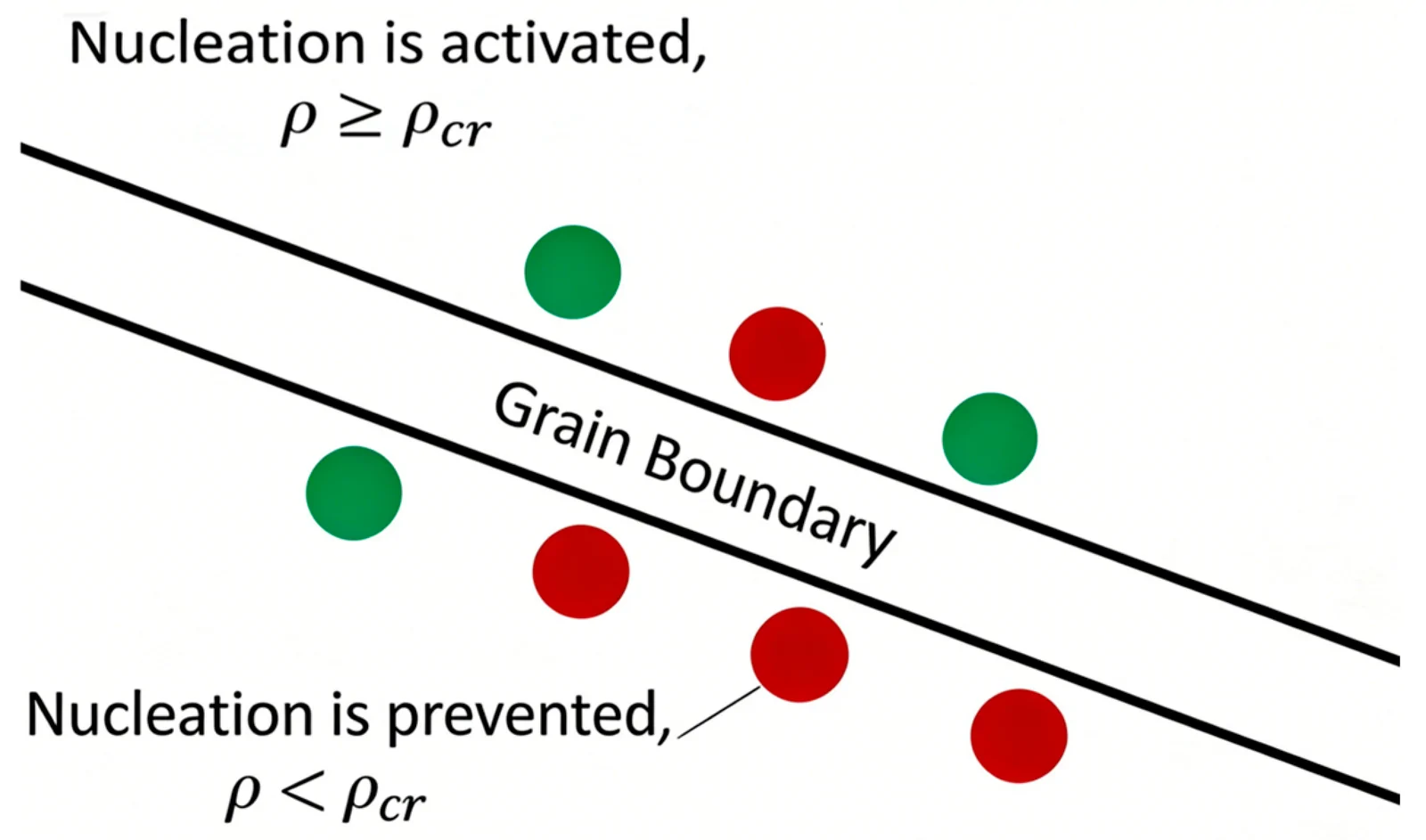
Schematic of grain nucleation process (***ρ***—dislocation density; ***ρ***_cr_—critical dislocation density; The red ones is prevented, and the green ones is activated).

**Table 1:** Initial grain size distribution of GH4706 alloy.

Grain Size (μm)	Volume Fraction	Grain Size (μm)	Volume Fraction
52.3565	0.01	161.8519	0.11
64.5232	0.03	186.1804	0.06
76.6899	0.05	198.3471	0.19
88.8566	0.03	210.5138	0.09
101.0233	0.01	222.6805	0.12
113.1851	0.02	234.8423	0.09
125.3518	0.04	247.009	0.07
149.6852	0.06		

**Table 2:** Process parameter field values and simulation results of research points.

Verification Points	Temperature (℃)	Strain Rate (s^−1^)	Effective Strain	*AVG* (μm)	*SD* (μm)
P1	868	0.015	0.24	142.12	23.61
P2	1130	0.032	2.19	26.71	1.35
P3	957	0.013	0.32	122.12	17.84
P4	868	0.010	0.44	81.56	14.33
P5	1140	0.026	2.05	23.89	1.07
P6	1020	0.011	0.64	52.35	4.64
P7	985	0.039	1.74	37.76	2.01
P8	1150	0.042	1.93	23.22	1.45
P9	1070	0.034	1.79	26.71	1.32
P10	1060	0.038	1.83	30.26	1.27
P11	1160	0.059	1.55	33.78	1.35
P12	1040	0.022	1.19	29.35	1.83
Integral Turbine disk 29.43 20.10				29.43	20.10

**Table 3:** Relative deviation between simulated and experimental results.

Objects	Verification Points	Experimental Value	Simulated Value	*Δ* (%)	*Δ_avg_* (%)	*ξ* (%)
*AVG* (μm)	P1	142.1	137.3	3.51	5.21	3.26
	P3	122.1	132.6	7.90		
	P4	81.6	83.7	2.56		
	P6	52.4	57.8	9.43		
	P10	30.3	32.4	6.60		
	P12	29.4	29.7	1.18		
*SD* (μm)	P1	23.6	22.0	7.46	9.65	4.06
	P3	17.84	18.35	2.78		
	P4	14.33	12.46	15.01		
	P6	4.64	5.15	9.90		
	P10	1.87	2.17	13.82		
	P12	1.83	1.68	8.93		
DRX fraction (%)	P1	24.1	27.3	11.65	5.31	3.12
	P3	22.1	22.6	6.27		
	P4	40.3	42.4	5.05		
	P6	39.4	41.7	3.40		
	P10	81.6	83.7	2.56		
	P12	90.4	87.8	2.90		

## Data Availability

The original contributions presented in this study are included in the article. Further inquiries can be directed to the corresponding authors.
